# Attitudes to Vaccine Mandates among Late Adopters of COVID-19 Vaccines in Zimbabwe

**DOI:** 10.3390/vaccines10071090

**Published:** 2022-07-07

**Authors:** Azure Tariro Makadzange, Patricia Gundidza, Charles Lau, Janan Dietrich, Norest Beta, Nellie Myburgh, Nyasha Elose, Chiratidzo Ndhlovu, Wilmot James, Lawrence Stanberry

**Affiliations:** 1 Charles River Medical Group, 155 King George Avenue, Avondale, Harare, Zimbabwe; patricia.gundidza@crmgresearch.com (P.G.); norest.beta@crmgresearch.com (N.B.); nyasha.elose@crmgresearch.com (N.E.); rati.ndhlovu@crmgresearch.com (C.N.); 2 RTI International, 3040 East Cornwallis Road, Research Triangle Park, Research Triangle, NC 27709, USA; clau@rti.org; 3 Perinatal HIV Research Unit (PHRU), Faculty of Health Sciences, University of the Witwatersrand, Johannesburg 2000, South Africa; dietrichj@phru.co.za; 4 African Social Sciences Unit of Research and Evaluation (ASSURE), a Division of the Wits Health Consortium, Faculty of Health Sciences, University of the Witwatersrand, Johannesburg 2000, South Africa; 5 Health Systems Research Unit, South African Medical Research Council, Bellville 7530, South Africa; 6 Wits Vaccines & Infectious Diseases Analytics (VIDA) Research Unit, Faculty of Health Sciences, University of the Witwatersrand, Johannesburg 2000, South Africa; nellie.myburgh@wits-vida.org; 7 Internal Medicine Unit, Faculty of Health Sciences, University of Zimbabwe, Harare, Zimbabwe; 8 Institute for Social and Economic Research and Policy, Columbia University, IAB 118th Street, New York, NY 10025, USA; wgj2104@columbia.edu; 9 Vaccine Information Network, Columbia University, 533 W 218th St., New York, NY 10032, USA; lrs2155@cumc.columbia.edu; 10 Department of Pediatrics, Vagelos College of Physicians and Surgeons, Columbia University, New York, NY 10032, USA

**Keywords:** vaccine mandates, vaccine hesitancy, COVID-19 vaccine

## Abstract

Despite sufficient supply, <25% of the population in sub-Saharan Africa has received at least one dose of COVID-19 vaccine. Vaccine mandates have previously been effective in increasing vaccine uptake. Attitudes to COVID-19 vaccine mandates and vaccines for children in African populations are not well understood. We surveyed late-adopters presenting for COVID-19 vaccination one year after program initiation in Zimbabwe. Logistic regression models were developed to evaluate factors associated with attitudes to mandates. In total, 1016 adults were enrolled; 690 (67.9%) approved of mandating vaccination for use of public spaces, 686 (67.5%) approved of employer mandates, and 796 (78.3%) approved of mandating COVID-19 vaccines for schools. Individuals of lower economic status were twice as likely as high-income individuals to approve of mandates. Further, 743 (73.1%) participants indicated that they were extremely/very likely to accept vaccines for children. Approval of vaccine mandates was strongly associated with perceptions of vaccine safety, effectiveness, and trust in regulatory processes that approved vaccines. Vaccine hesitancy is an important driver of low vaccine coverage in Africa and can be mitigated by vaccine mandates. Overall, participants favored vaccine mandates; however, attitudes to mandates were strongly associated with level of education and socioeconomic status.

## 1. Introduction

Novel coronavirus infections have led to significant regional or global pandemics over the last 20 years [1,2]. The severe acute respiratory syndrome coronavirus (SARS-CoV) pandemic in 2003 resulted in 774 deaths, and the ongoing Middle East Respiratory Syndrome has resulted in at least 850 deaths [3]. The SARS-CoV-2 pandemic has, to date, resulted in over 500 million infections and 6 million deaths [4]. Within a year of sequence identification, highly effective vaccines against SARS-CoV-2 were developed, and the World Health Organization had given emergency use listing for 10 vaccines across several different platforms by the end of 2021 [5]. Despite the presence of effective and safe vaccines, globally only 67.2% of eligible people have been vaccinated. In Africa, only 23.5% of the population has received at least one dose of vaccine [6]. As the supply of vaccines has improved globally, it is increasingly evident that vaccine hesitancy is contributing to low vaccine uptake in Africa [7]. Vaccine hesitancy is defined as a ‘delay in acceptance or refusal of vaccination despite the availability of vaccination services’ [8]. Voluntary vaccination may not be sufficient to achieve vaccination coverage goals, and vaccine mandates may be necessary [9,10,11,12].

In Zimbabwe, by June 2022, only 50% of the population had received at least one dose of a COVID-19 vaccine despite the availability of COVID-19 vaccines for more than a year [6]. An online survey conducted in February 2021 in Zimbabwe just before vaccines became widely available, found that 49.9% of the study population would voluntarily receive the COVID-19 vaccine, with young adults (18–25 years) having the lowest vaccine acceptance rate [13]. Most of the population lacked confidence in the safety of the vaccine and half distrusted the government’s ability to ensure the safety of the vaccine [13]. 

Childhood vaccine mandates have been in place for decades in many countries in Africa and the developed world, resulting in high childhood vaccination coverage rates [14]. Similarly, mandates for influenza vaccination of healthcare workers led to significant increase in vaccination uptake [15]. The low uptake in COVID-19 vaccinations has led several governments and employers to consider the introduction of vaccine mandates. Major companies have mandated vaccines, particularly for consumer-facing staff [16]. African governments have also followed similar approaches [17]. The government of Zimbabwe introduced a COVID-19 vaccination mandate for public employees in 2021. The impact of these employment vaccine mandates may be minimal and hard to measure, particularly in areas with high rates of informal employment [18]. Mandates related to use of public facilities, such as transportation services, or childhood vaccine mandates could be potentially more impactful. 

We evaluated the attitudes to vaccine mandates and vaccines for children in a cohort of adults who were presenting at public vaccination centers in Harare, Zimbabwe. The cohort consists of individuals who were presenting for their first vaccine dose almost 12 months after the public program for free COVID-19 vaccination was initiated. These individuals lie on the vaccine hesitancy spectrum, choosing to ‘wait and see’ for several months prior to accepting vaccination [19]. Studying late vaccine adopters provides insight into ongoing barriers and motivations for vaccination. As uptake rates within vaccine programs stall in Africa, mandates may become important for driving late adopters from the sidelines into the vaccination centers. 

## 2. Materials and Methods

### 2.1. Study Design

Between 4 January and 11 February 2022, we conducted a quantitative survey to evaluate attitudes, barriers, motivations, key influencers, and information sources for vaccination among individuals presenting for COVID-19 vaccines in public vaccination centers in Harare [20]. 

### 2.2. Study Sites and Sampling

Adults (age >18 years) who were receiving their first dose of COVID-19 vaccines at public sites at 5 City of Harare clinics, including their affiliated outreach sites (Mabelreign, Avondale, Belvedere, and Dzivarasekwa), were consecutively enrolled into the study. All individuals receiving their first COVID-19 vaccine were eligible to participate, except those who had obvious cognitive impairments or were unable to provide informed consent.

A comprehensive questionnaire was prepared as previously described [20]. The questionnaire was administered in the language preferred by the participant, either English or Shona. Each participant received USD 5 as compensation. The questionnaire consisted of six main sections: demographics, attitudes and views towards COVID-19 vaccines, barriers, motivations, information sources, and vaccination experience. Attitudes towards vaccine mandates and vaccines for children were assessed (Table 1). Following receipt of the first vaccination dose, consenting participants were interviewed by a member of the survey team. All study data were confidential. 

### 2.3. Statistical Analysis

Data for the categorical questions were described using absolute numbers, proportions, and percentages.

Chi-squared tests were used to compare responses for different sub-groups, e.g., gender, HIV status, economic status, and educational level. Statistical significance was assessed using *p*-values. A multiple logistic regression analysis with backward selection was used to identify independent predictors of vaccine mandates. Factors associated with ‘strongly approve’ and ‘somewhat approve’ of the mandates were examined. For this analysis, the outcome responses were dichotomized into two categories (strongly approve/does not strongly approve or yes/no). Based on a priori considerations, the variables evaluated included demographic factors such as gender, education, a proxy for income that was defined by availability of alternate sources of energy (battery or generator), and/or refrigerator ownership, personal experience with COVID-19—knowing someone who became severely ill or died from COVID-19, use of internet in the past 30 days, WhatsApp or Facebook use; and the presence of children below 18 years of age in the household; HIV status. We also included the analysis of responses to four questions on perceived safety and effectiveness of COVID-19 vaccines. Responses to questions assessing perceived safety of COVID-19 vaccines, confidence in regulatory processes, and effectiveness of responses were dichotomized into two categories: Agree (strongly agree/somewhat agree) and Disagree. Responses to the perceived safety of inactivated whole virus vaccines (Sinovac and Sinopharm) were dichotomized into Safe (very safe/somewhat safe) and Unsafe. Participants were classified as high socioeconomic status if they owned a refrigerator and used a battery or generator for power, low economic status if they had neither a refrigerator nor a battery or generator for power; the rest were classified as middle economic status. The strength of the investigated associations was described using adjusted odds ratios (ORs) and the corresponding 95% confidence intervals and variables with a *p*-value ≤ 0.05 remained in the final model (two-sided). Analyses were performed using IBM SPSS Statistics^®^ version 23 (IBM Corp., Armonk, NY, USA). Multicollinearity was assessed by computing the variance inflation factor (VIF). *p*-values were two-sided and 0.05 was used as the level of confidence.

### 2.4. Ethics Approvals

The protocol, consent forms, and recruitment materials were reviewed and approved by the Medical Research Council of Zimbabwe (MRCZ), Joint Research Ethics Committee of Parirenyatwa Hospital, and the University of Zimbabwe (JREC) and Harare City Health Departments prior to initiation of the study. The MRCZ approval number was MRCZ/A/2809. The JREC approval number was JREC/373/2021. All amendments to the protocol, consent forms, and/or recruitment materials were approved by these institutional review boards before they were implemented. All participants provided written informed consent.

## 3. Results

We enrolled 1016 adults presenting for vaccination approximately one year after the initiation of the vaccination program as described [20]. Half of the cohort was female; the median age was 30 years (IQR: 22–39 years) (Table 2). Most participants had secondary or higher education; 5 (0.5%) had no education and 84 (8.3%) had only completed primary education. To classify their socioeconomic status, participants were asked about their energy sources or if they owned certain household items. Homes had electricity for 892 (87.7%), a refrigerator for 770 (75.8%), a television for 900 (88.6%), a bed for 990 (97.4%), and a battery or generator for power for 240 (23.8%) participants. Among participants, 420 (41.4%) indicated that they had used the internet in the last 30 days; 126 (12.4%) were people living with HIV infection (PLWH), 428 (42.1%) knew someone who had become severely ill or died from COVID-19 disease, and 866 (85.2%) lived in a household with children under the age of 18 years (Table 2). All participants were vaccinated with the Sinopharm vaccine, except for three participants who received the Sinovac vaccine. Perceptions of vaccine safety, effectiveness, and trust in regulatory processes were high (Table 2) [20].

**Figure 1:**
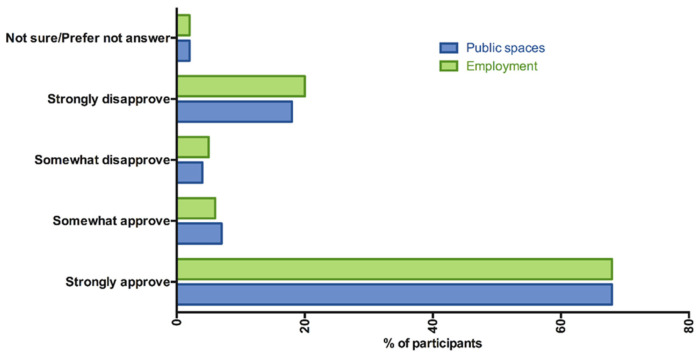
Attitudes to adult vaccine mandates. Study participants were asked: “Do you approve of the government requiring COVID-19 vaccine to get on public transportation?” or “Do you approve employers requiring a COVID-19 vaccine to get a job or certain pay allowances?”.

We evaluated attitudes to vaccine mandates by asking questions relating to vaccination requirements for use of public spaces e.g., public transportation, employment and employee benefits and school vaccination (Table 1). Among study participants, 690 (67.9%) participants strongly approved of the government requiring COVID-19 vaccination to get on public transportation (Figure 1). There was a trend of decreasing likelihood to strongly/somewhat approve of a mandate for public transportation with increasing education level (Table 3). Participants of low economic status were twice as likely to approve of the vaccination for public transport mandate than those of higher economic status (OR 2.25; 95% CI: 1.4, 3.61). Individuals who were confident in the regulatory process (OR 1.76: 95% CI 1.18, 2.61), effectiveness of vaccines in preventing disease (OR 2.35: 95% CI 1.54, 3.58), and who perceived the Sinovac/Sinopharm vaccines as safe (OR 1.84: 95% CI 1.35, 2.51) were twice as likely to strongly/somewhat approve of a vaccination mandate for use of public transportation.

When asked if employers should require COVID-19 vaccination as a condition for employment and or access to certain pay benefits, 686 (67.5%) strongly approved (Figure 1). Those with higher levels of education and particularly tertiary education were less likely to support employer mandates (Table 4). Those of a low economic status were almost twice as likely to strongly approve of an employment mandate than those of a higher economic status (OR 1.92; 95% CI: 1.21, 3.07) (Table 4). There were strong associations between confidence in regulatory process (OR 1.76: 95% CI 1.19, 2.62), effectiveness of vaccines in preventing disease (OR 3.2: 95% CI 2.11, 4.86), and perceiving the Sinovac/Sinopharm vaccines as safe (OR 1.95: 95% CI 1.43, 2.66) and strongly/somewhat approving of an employment mandate.

Among the study participants, 454 (44.7%) indicated that a reason to present for vaccination today was “To get back to work or school”. Among these participants, 322 (70.9%) indicated that it was a requirement from their employer or school that they should be vaccinated, 122 (26.9%) said it was not, and 10 (2.2%) did not know if it was required. Participants for whom vaccination was a requirement from their employer or school were less likely to approve of the government mandating vaccination for the use of public transportation (OR 0.69; 95% CI: 0.52, 0.92) or for employers mandating vaccination for employment or pay allowances (OR 0.69; 95% CI 0.53, 0.92).

Most participants lived in a household with school-age individuals below the age of 18 years (Table 2). Requiring students to receive COVID-19 vaccines by schools was highly favored; 796 (78.3%) indicated that schools should require COVID-19 vaccines. There was an association between increasing education level and internet use with not approving of a school mandate (Table 5). When restricting the analysis to only those with children below the age of 18 years at home, participants of a lower economic status (OR 1.93; 95% CI: 1.2, 3.12) and those with a personal experience with COVID-19 disease (OR 1.38; 95% CI: 1.003, 1.91) were more likely to agree to a school mandate. There were strong associations between confidence in the regulatory process for approving vaccines (OR 1.89: 95% CI 1.27, 2.83), effectiveness of vaccines in preventing disease (OR 1.71: 95% CI 1.11, 2.64) and perceiving the Sinovac/Sinopharm vaccines as safe (OR 1.75: 95% CI 1.27, 2.42) with approval of a vaccine mandate for school children. (Table 5).

**Figure 2:**
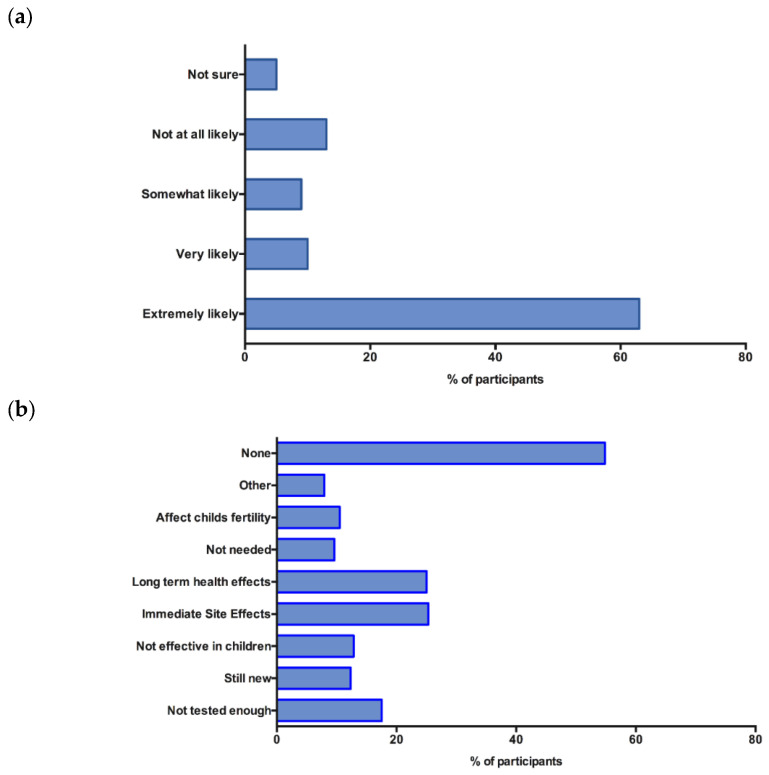
Attitudes towards COVID-19 vaccines for children. (**a**) Likelihood of vaccinating children. Participants were asked “If the COVID-19 vaccine was available to your child, how likely would you be to get your child vaccinated” (**b**) Concerns about vaccines for children, participants were asked “What concerns about COVID-19 vaccines for do you have, if any?”.

At the time the survey had been developed, vaccines had not been made available for children younger than 15 years. We asked participants how likely they would be to vaccinate their child should the COVID-19 vaccine be made available; 743 (73.1%) participants indicated that they would be extremely or very likely to get their children vaccinated. Among the study participants, 557 (54.8%) had no concerns about childhood vaccines, 257 (25.3%) were concerned about immediate side effects, 254 (25%) were concerned about long-term health effects, and 178 (17.5%) were concerned that the vaccine had not been tested enough in children (Figure 2). Several other concerns were raised, such as the limited experience with the vaccines in children, limited trust, and wanting to ‘first see how other children respond to the vaccine’.

Those with children in the home were twice as likely to indicate that they would get their children vaccinated (OR 2.09; 95% CI: 1.42, 3.09) (Table 6). Lower socioeconomic status was also associated with increased likelihood of intent to vaccine their children. There were strong associations between intention to get children vaccinated and confidence in COVID-19 vaccine safety (OR 2.02: 95% CI 1.29, 3.18), regulatory process (OR 1.76: 95% CI 1.17, 2.67), effectiveness of vaccines in preventing disease (OR 1.64: 95% CI 1.03, 2.60), and perceiving the Sinovac/Sinopharm vaccines as safe (OR 1.8: 95% CI 1.31, 2.47), (Table 6). 

## 4. Discussion

Vaccine mandates can be an effective way to improve vaccine coverage rates and counter vaccine hesitancy. Vaccine hesitancy, coupled with healthcare systems inadequate infrastructure to deliver vaccines, is increasingly playing an important role in low vaccine coverage in Africa [6,21]. Studies conducted early in the course of the pandemic suggested uptake rates would be high, while hesitancy would be driven by a variety of factors including concerns about side effects, vaccine-associated myths circulated on social media, and complacency due to risk perception [22,23]. Attitudes vary across the continent with higher vaccine acceptance rates in Eastern and Southern Africa compared with West Africa [7]. Vaccine mandates historically have been an important tool in improving vaccine uptake rates [24]. Attitudes to mandates are well described in western cohorts with limited data in African populations [25]. We evaluated attitudes towards vaccine mandates in an African cohort composed of individuals who decided to accept vaccination one year after initiation of the national vaccine program. These are neither early vaccine adopters nor hard-core resisters and represent a large “middle segment” in society that may be important in determining support for mandates in the large remaining segment of unvaccinated individuals.

We assessed the acceptance of vaccination requirements for access to public spaces, e.g., public transport, for employment and employment benefits and for school. The acceptance of mandates was high, with 70% strongly or somewhat approving vaccine mandates for use of public transport or employment and employment benefits. This contrasts with some data from western nations that suggest lower mandate acceptance rates, that are often driven by factors such as age, gender, political affiliations, perceptions of vaccine safety and effectiveness, and trust in regulatory processes [25,26,27]. Recent data from Columbia, El Salvador, and Spain show high rates of support of mandates, but low support for active measures such as suspension from work for those refusing vaccination [28]. The high acceptance rates for mandates that we observed was driven by socioeconomic status and perceptions of vaccine safety, effectiveness, and trust in government regulatory processes. Age, gender, personal experience with COVID-19, and presence of co-infections such as HIV infection were not associated with acceptance of mandates. We saw a trend of those with increased education level having less support of mandates. Building confidence around vaccine safety and effectiveness and strengthening trust in regulatory process for vaccine approval will be critical for reducing vaccine hesitancy and acceptance of mandates particularly in groups with higher educational attainment and socioeconomic status. 

The introduction of mandates needs to be cautiously considered. Individual rights need to be balanced with collective communal rights and public health responsibilities. Perceptions of violation of individual rights and government overreach can result in significant sociopolitical backlash. We observed that individuals who indicated that they came to be vaccinated because it was required were less likely to accept mandates than those for whom no requirement was in place. This is similar to observational data in the UK that assessed views on vaccine mandates and identified key themes, including that although mandates may be necessary, they may be perceived as over-reach of state power, remove autonomy in decision making, and may create ‘vaccine apartheid’ [12]. Policy makers in Africa will have to tread carefully, balancing public and individual rights as they consider introducing vaccine mandates.

Attitudes to vaccine mandates for school attendance were slightly more favorable, with almost 80% of the study population agreeing with COVID-19 vaccine mandates for schools and over 70% indicating that they would be extremely or very likely to vaccinate their children. The greatest concerns regarding vaccines for children were around short- and long-term side effects and were similar to trends in concerns about COVID-19 vaccines for adults [20]. Approval of vaccine mandates for children was also associated with low economic status, and confidence in vaccine safety, effectiveness, and trust in regulatory processes. A trend towards a lower likelihood to support vaccine mandates for children was observed in those using the internet, WhatsApp, and Facebook. Increasing access to social media and associated misinformation and disinformation can play an important role in shaping vaccine hesitancy [29]. As more people in Africa use the internet as an important source of health information, public health officials will likely have to deal with increasing levels of vaccine hesitancy due to misinformation. 

Our data contrast with similar data from the United States. In a web-based survey conducted in the United States, 48.6% found vaccination for children attending school as acceptable, 40.9% found state mandates for vaccination acceptable, and 47.7% found employer-enforced employee mandates acceptable [30]. Differences in attitudes to mandates fell across racial, educational, and political lines. Those with tertiary education were more likely to find mandates acceptable than those without [30]. In our cohort, mandate acceptance rates were higher and wealth was associated with decreased acceptability of vaccine mandates including COVID-19 vaccines for children. An online survey conducted in Zimbabwe just prior to the availability of COVID-19 vaccines supports high levels of vaccine hesitancy in Zimbabwe, particularly in individuals of higher socioeconomic status with internet access [13]. Half of the participants indicated that they would not undergo voluntary vaccinations and most indicated that they had concerns about vaccine safety, effectiveness, and trust in government regulatory processes [13]. The survey was an internet-based survey and almost all had tertiary education. Wealth, education, and internet use are likely to become future important drivers of vaccine hesitancy and resistance to mandates in Africa particularly as family members of higher education and socioeconomic status often have disproportionately large influence on decision-making. This is important for vaccines as our previous data indicated that key influencers for vaccination were friends and family [20]. Public health officials in Africa will need to proactively address the concerns of this growing cohort of educated internet users who may start to play an important role in undermining the previously high levels of vaccine confidence in the region [31]. 

## 5. Strengths and Limitations

Our study has certain limitations. Our survey was of individuals who transitioned from a ‘wait and see’ status to acceptance. For a significant proportion of the study population, the decision to be vaccinated was driven by a workplace or a school requirement. The study does not capture the attitudes of those who continue to not accept vaccination. There is evidence of high levels of vaccine hesitancy in Zimbabwe, and future studies will need to focus on those that remain unvaccinated. Our questions on mandates focused on use of public facilities and employment as these are requirements that the government of Zimbabwe had instituted; however, we did not address more targeted mandates such as mandates for healthcare workers. Our income categorization was based on assets and energy sources, which are useful parameters particularly in settings where income is fluid and formal employment are low [18]. However, dollar-based income categorization would have provided further granularity on the income status of the cohort. This is particularly important given the evidence of the role of internet use and access to WhatsApp and Facebook have on attitudes to vaccines. 

However, the study has a number of key strengths, including that it was an in-person survey conducted after vaccines had been available for almost a year. Vaccine convenience had largely been addressed while matters of confidence persisted [20]. Many studies of COVID-19 vaccine hesitancy and attitudes to mandates have focused on online surveys and were conducted either before vaccine availability or soon after availability where predicting intention vs. actual behavior would have been challenging [13,22]. Internet-based surveys are useful in countries with high internet penetration and low data costs. The World Bank estimates that only 29% of the population in Zimbabwe use internet with similar estimates in other African countries [32]. Our study provides insights into a significant proportion of the urban African population that may not have regular internet access.

## 6. Conclusions

The study data presented evaluated attitudes towards vaccination mandates for use of public spaces, employment, and school attendance in a cohort of late vaccine adopters. Acceptance of mandates was approximately 70% for use of public spaces, employment, and almost 80% for school attendance. The main factors associated with acceptance of mandates was lower socioeconomic status and higher levels of perceived vaccine safety, effectiveness, and trust in government regulatory processes that approved vaccines. 

The acceptability of employer-enforced mandates points to a potential role for employers to increase COVID-19 vaccine uptake. The acceptability of mandates to use public spaces may play an important role, particularly in settings of high informal employment, but may be difficult to enforce. Vaccine mandates will likely be acceptable in many places in Africa; however, the implementation of mandates should be coupled with strong efforts to address concerns and increase confidence in vaccine safety, effectiveness, and regulatory processes for vaccine approval.

## Figures and Tables

**Table 1:** Survey questions addressing vaccine mandates.

Question	Response Options
Attitudes to vaccine Mandates	
Do you approve of the government requiring a COVID-19 vaccine to get on public transportation?	Strongly approve; Somewhat approve; Somewhat disapprove; Strongly disapprove; Not sure; Prefer not to answer
Do you approve employers requiring a COVID-19 vaccine to get a job or certain pay allowances?	Strongly approve; Somewhat approve; Somewhat disapprove; Strongly disapprove; Not sure; Prefer not to answer
Do you think schools should require students to be vaccinated for COVID-19 as they do for most other diseases like measles and tuberculosis?	Yes, schools should require COVID-19 vaccination; No, schools should not require COVID-19 vaccination
Was there a requirement from your employer or school that you be vaccinated?	Yes, No, Don’t know
Attitudes to Childhood Vaccines	
If the COVID-19 vaccine was available to your child, how likely would you be to get your children vaccinated?	Extremely likely; Very likely; Somewhat likely; Not at all likely; Not sure
What concerns about COVID-19 vaccines for children do you have, if any? (Multiple responses accepted)	The vaccine has not been tested enough in children; the vaccine is still new; The vaccine is not effective in children; Immediate side effects; Long-term health effects; children do not need the vaccine because COVID-19 is mild in children; COVID-19 vaccines affect children’s fertility in the future; Other (specify); I have no concerns
Perceptions of vaccine safety and efficacy	
In general, COVID-19 vaccines are safe	Strongly agree; Somewhat agree; Neutral; Somewhat disagree; Strongly disagree; I do not know; Prefer not to answer
I am confident that my country’s regulation process approved the COVID-19 vaccine, only when it was shown to be safe	Strongly agree; Somewhat agree; Neutral; Somewhat disagree; Strongly disagree; I do not know; Prefer not to answer
I am confident that COVID-19 vaccines are effective in preventing the disease	Strongly agree; Somewhat agree; Neutral; Somewhat disagree; Strongly disagree; I do not know; Prefer not to answer
How safe or unsafe is the Sinovac/Sinopharm vaccine?	Very safe; Somewhat safe; Somewhat unsafe; Very unsafe; I do not know; Prefer not to answer

**Table 2:** Baseline cohort demographics and perceptions of vaccine safety, effectiveness, and trust in regulatory processes of study cohort (*n =* 1016).

Characteristic	N(%)
Gender (Female)	508 (50%)
Median age (IQR)	30 (22–39)
Age groups	
18–25	368 (36.2%)
26–39	409 (40.3%)
>=40 years	239 (23.5%)
Ethnicity (Black African)	1016 (100%)
Highest Level of Education	
None + primary	89 (8.8%)
Lower secondary	735 (72.4%)
Higher secondary	117 (11.5%)
Tertiary	74 (7.3%)
Co-morbid conditions	
HIV	126 (12.4%)
Socioeconomic status	
Higher	172 (16.9 %)
Middle	598 (58.9%)
Lower	246 (24.2%)
Internet use in past 30 days (Y)	420 (41.4 %)
Personal knowledge of someone who was seriously ill/died from COVID-19	428 (42.1%)
Children under age of 18 in the household (Y)	866 (85.2%)
In general, COVID-19 vaccines are safe (strongly + somewhat agree)	856 (84.3%)
I am confident that my country’s regulation process approved the COVID-19 vaccine only when it was shown to be safe (strongly + somewhat agree)	838 (82.5%)
I am confident that COVID-19 vaccines are effective in preventing the disease (strongly+somewhat agree)	868 (85.4%)
How safe or unsafe is the Sinovac/Sinopharm vaccine? (very + somewhat safe)	675 (66.4%)

**Table 3:** Do you approve of the government requiring a COVID-19 vaccine to use public transportation? (strongly/somewhat agree).

			Univariate			Multivariate		
Variables	Category	Proportion That Approves	OR *	95% CI ^#^	*p*-Value	OR *	95% CI ^#^	*p*-Value
**Gender**	Male	0.75	1					
	Female	0.75	1.021	0.769, 1.357	0.885			
**Age**	18–25	0.76	1					
	26–39	0.75	0.944	0.679, 1.312	0.732			
	≥40 years	0.72	0.812	0.56, 1.177	0.271			
**Education**	≤primary	0.83	1					
	Lower secondary	0.76	0.644	0.360, 1.150	0.137			
	Higher secondary	0.73	0.538	0.271, 1.071	0.078			
	Tertiary	0.58	0.281	0.137, 0.0579	0.001			
**Economic status**	High	0.65	1					
	Middle	0.75	1.6	1.112, 2.303	0.011	1.588	1.083, 2.329	0.018
	Low	0.82	2.459	1.564, 3.866	<0.001	2.249	1.403, 3.607	0.001
**Personal COVID Experience**	No	0.73	1					
	Yes	0.78	1.328	0.992, 1.779	0.057			
**HIV**	Negative	0.75	1					
	Positive	0.74	0.931	0.608, 1.424	0.742			
**Internet use in last 30 days**	No	0.78	1					
	Yes	0.71	0.684	0.514, 0.912	0.01			
**Children at home**	No	0.74	1					
	Yes	0.75	1.064	0.716, 1.581	0.759			
**WhatsApp**	No	0.77	1					
	Yes	0.74	0.816	0.606, 1.098	0.179			
**Facebook**	No	0.77	1					
	Yes	0.72	0.796	0.595, 1.065	0.124			
**In general, COVID-19 vaccines are safe**	Disagree	0.53	1					
	Agree	0.79	3.337	2.348, 4.743	<0.0001			
**I am confident that my country’s regulation process approved the COVID-19 vaccine, only when it was shown to be safe**	Disagree	0.56	1					
	Agree	0.79	2.934	2.089, 4.12	<0.0001	1.756	1.183, 2.605	0.005
**I am confident that COVID-19 vaccines are effective in preventing the disease**	Disagree	0.5	1					
	Agree	0.79	3.822	2.662, 5.487	<0.0001	2.349	1.543, 3.576	<0.0001
**How safe or unsafe is the Sinovac/Sinopharm vaccine?**	Unsafe	0.64	1					
	Safe	0.8	2.292	1.711, 3.068	<0.0001	1.838	1.345, 2.511	<0.0001

* OR = Odds ratio. ^#^ CI = Confidence interval.

**Table 4:** Do you approve employers requiring a COVID-19 vaccine to get a job or certain pay allowances? (Strongly/somewhat agree).

			Univariate			Multivariate		
Variables	Category	Proportion That Approves	OR *	95% CI ^#^	*p*-Value	OR *	95% CI ^#^	*p*-Value
**Gender**	Male	0.71	1					
	Female	0.76	1.261	0.954, 1.667	0.103			
**Age**	18–25	0.74	1					
	26–39	0.74	1.009	0.732, 1.391	0.957			
	≥40 years	0.71	0.87	0.604, 1.251	0.452			
**Education**	≤primary	0.84	1					
	Lower secondary	0.75	0.547	0.302, 0.991	0.047			
	Higher secondary	0.67	0.373	0.188, 0.743	0.005			
	Tertiary	0.58	0.259	0.124, 0.539	<0.001			
**Economic status**	High	0.66	1					
	Middle	0.72	1.359	0.946, 1.952	0.097	1.332	0.904, 1.962	0.148
	Low	0.81	2.27	1.449, 3.558	<0.001	2.109	1.31, 3.395	0.002
**Personal COVID Experience**	No	0.72	1					
	Yes	0.76	1.21	0.911, 1.608	0.188			
**HIV**	Negative	0.73	1					
	Positive	0.75	1.132	0.735, 1.742	0.575			
**Internet use in last 30 days**	No	0.77	1					
	Yes	0.68	0.621	0.469, 0.822	0.01			
**Children at home**	No	0.73	1					
	Yes	0.73	1	0.675, 1.479	0.998			
**WhatsApp**	No	0.76	1					
	Yes	0.72	0.797	0.596, 1.067	0.128			
**Facebook**	No	0.75	1					
	Yes	0.7	0.758	0.571, 1.008	0.057			
**In general, COVID-19 vaccines are safe**	Disagree	0.52	1					
	Agree	0.77	3.166	2.233, 4.488	<0.0001			
**I am confident that my country’s regulation process approved the COVID-19 vaccine, only when it was shown to be safe**	Disagree	0.52	1					
	Agree	0.78	3.204	2.289, 4.484	<0.0001	1.766	1.192, 2.615	0.005
**I am confident that COVID-19 vaccines are effective in preventing the disease**	Disagree	0.42	1					
	Agree	0.79	5.121	3.556, 7.375	<0.0001	3.201	2.11, 4.855	<0.0001
**How safe or unsafe is the Sinovac/Sinopharm vaccine?**	Unsafe	0.61	1					
	Safe	0.8	2.542	1.908, 3.387	<0.0001	1.952	1.434, 2.656	<0.0001

* OR = Odds ratio. ^#^ CI = Confidence interval.

**Table 5:** Do you think schools should require students to be vaccinated for COVID-19 as they do for most other diseases such as measles and tuberculosis? (Yes; analysis of only those who indicated that they have children below age 18 years in the household).

			Univariate			Multivariate		
Variables	Category	Proportion That Approves	OR *	95% CI ^#^	*p*-Value	OR *	95% CI^#^	*p*-Value
**Gender**	Male	0.78	1					
	Female	0.8	1.129	0.816, 1.562	0.463			
**Age**	18–25	0.8	1					
	26–39	0.79	0.945	0.65, 1.375	0.769			
	>=40 years	0.76	0.821	0.533, 1.265	0.371			
**Education**	≤primary	0.82	1					
	Lower secondary	0.79	0.851	0.453, 1.599	0.616			
	Higher secondary	0.83	1.121	0.506, 2.483	0.779			
	Tertiary	0.59	0.327	0.148, 0.724	0.006			
**Economic status**	High	0.7	1					
	Middle	0.79	1.63	1.072, 2.48	0.022	1.589	1.071, 2.358	0.021
	Low	0.83	2.027	1.222, 3.363	0.006	1.931	1.195, 3.121	0.007
**Personal COVID Experience**	No	0.76	1					
	Yes	0.83	1.551	1.102, 2.183	0.012	1.384	1.003, 1.909	0.048
**HIV**	Negative	0.79	1					
	Positive	0.78	0.972	0.593, 1.592	0.91			
**Internet use in last 30 days**	No	0.81	1					
	Yes	0.75	0.7	0.505, 0.97	0.032			
**WhatsApp**	No	0.82	1					
	Yes	0.76	0.701	0.495, 0.992	0.045			
**Facebook**	No	0.81	1					
	Yes	0.75	0.68	0.489, 0.944	0.021			
**In general, COVID-19 vaccines are safe**	Disagree	0.62	1					
	Agree	0.81	2.701	1.88, 3.881	<0.0001			
**I am confident that my country’s regulation process approved the COVID-19 vaccine, only when it was shown to be safe**	Disagree	0.62	1					
	Agree	0.82	2.79	1.967, 3.958	<0.0001	1.893	1.266, 2.831	0.002
**I am confident that COVID-19 vaccines are effective in preventing the disease**	Disagree	0.6	1					
	Agree	0.82	2.911	2.009, 4.218	<0.0001	1.709	1.105, 2.643	0.016
**How safe or unsafe is the Sinovac/Sinopharm vaccine?**	Unsafe	0.7	1					
	Safe	0.83	2.064	1.521, 2.801	<0.0001	1.751	1.265, 2.424	0.001

* OR = Odds ratio. ^#^ CI = Confidence interval.

**Table 6:** If the COVID-19 vaccine was available to your child, how likely would you be to get your child vaccinated? (Extremely likely/Very Likely).

			Univariate			Multivariate		
Variables	Category	Proportion of Participants	OR *	95% CI ^#^	*p*-Value	OR *	95% CI ^#^	*p*-Value
**Gender**	Male	0.71	1					
	Female	0.75	1.21	0.916, 1.598	0.179			
**Age**	18–25	0.66	1			1		
	26–39	0.78	1.819	1.325, 2.499	<0.001	1.608	1.14, 2.267	0.007
	≥40 years	0.77	1.701	1.177, 2.46	0.005	1.694	1.126, 2.55	0.011
**Education**	≤Primary	0.79	1					
	Lower secondary	0.75	0.819	0.48, 1.396	0.463			
	Higher secondary	0.68	0.587	0.31, 1.112	0.102			
	Tertiary	0.54	0.319	0.161, 0.632	0.001			
**Economic status**	High	0.63	1					
	Middle	0.74	1.724	1.203, 2.469	0.003	1.568	1.062, 2.314	0.024
	Low	0.77	2.011	1.309, 3.089	0.001	1.433	0.896, 2.293	0.134
**Personal COVID Experience**	No	0.72	1					
	Yes	0.74	1.109	0.836, 1.47	0.473			
**HIV**	Negative	0.73	1					
	Positive	0.76	1.202	0.777, 1.858	0.408			
**Internet use in last 30 days**	No	0.77	1			1		
	Yes	0.67	0.589	0.445, 0.779	<0.001	0.71	0.519, 0.971	0.032
**Children at home**	No	0.6	1			1		
	Yes	0.75	2.044	1.424, 2.934	<0.001	2.091	1.417, 3.086	<0.0001
**WhatsApp**	No	0.73	1					
	Yes	0.73	0.975	0.732, 1.299	0.863			
**Facebook**	No	0.73	1					
	Yes	0.73	1.017	0.62, 1.355	0.911			
**In general COVID-19 vaccines are safe**	Disagree	0.48	1			1		
	Agree	0.78	3.901	2.749, 5.534	<0.0001	2.021	1.287, 3.175	0.002
**Confident in regulatory process that approved the vaccines**	Disagree	0.52	1			1		
	Agree	0.78	3.254	2.326, 4.554	<0.0001	1.762	1.165, 2.666	0.007
**Confident that vaccines are effective in preventing the disease**	Disagree	0.47	1			1		
	Agree	0.78	3.846	2.683, 5.512	<0.0001	1.636	1.030, 2.598	0.037
**How safe or unsafe is the Sinovac/Sinopharm vaccine?**	Unsafe	0.62	1			1		
	Safe	0.79	2.244	1.686, 2.987	<0.0001	1.795	1.305, 2.467	<0.0001

* OR = Odds ratio. ^#^ CI = Confidence interval.

## Data Availability

The data presented in this publication will be openly available in a repository with DOI number at the time of publication.
